# Analysis of Postoperative Bleeding After Oral Surgery in Patients Receiving Anticoagulants: A Retrospective Study

**DOI:** 10.3390/medicina61030425

**Published:** 2025-02-28

**Authors:** Jae-Il Lee, Hyejun Seo, Yeong-Cheol Cho, Jang-Ho Son, Iel-Yong Sung

**Affiliations:** 1 Department of Dentistry, University of Ulsan College of Medicine, Ulsan University Hospital, Ulsan 44033, Republic of Korea; wodlf5887@nate.com (J.-I.L.); herrjoon@uuh.ulsan.kr (H.S.); 2 Department of Oral and Maxillofacial Surgery, University of Ulsan College of Medicine, Ulsan University Hospital, Ulsan 44033, Republic of Korea; 0729211@uuh.ulsan.kr (Y.-C.C.); ribosome@hanmail.net (J.-H.S.)

**Keywords:** bleeding, oral anticoagulants, oral surgery, warfarin, non-vitamin K oral anticoagulants

## Abstract

*Background and Objectives*: Patients taking anticoagulants, particularly warfarin and non-vitamin K oral anticoagulants (NOACs), face an elevated risk of postoperative bleeding during minor oral surgeries, highlighting the urgent need to identify reliable predictors for bleeding complications. In this study, we evaluated the effectiveness of predictors of bleeding complications in patients receiving anticoagulants who underwent minor oral surgeries. *Materials and Methods*: The electronic medical and dental records of 206 patients who underwent oral surgery at the University of Ulsan Hospital between 2015 and 2023 were retrospectively reviewed. Patients were categorized into those taking warfarin and those taking NOACs, and postoperative bleeding was determined. Risk factors were statistically analyzed using the chi-square or Fisher’s exact test and Student’s t-test. *Results*: Among the 206 patients (86 on warfarin, 120 on NOACs), 84 (36 on warfarin, 48 on NOACs) experienced bleeding complications following their procedures. Time in the therapeutic range (TTR) and international normalized ratio (INR) values were significantly associated with bleeding complications in the warfarin group, while the type of NOAC was associated with bleeding in the NOAC group. Perioperative bleeding was significantly correlated with postoperative bleeding in both groups. *Conclusions*: Taken together, these findings highlight the correlations between postoperative bleeding and specific factors associated with anticoagulant drugs in patients that underwent oral surgery. Identifying these predictors can improve patient management by enhancing pre- and perioperative assessments, reducing the risk of bleeding, and optimizing surgical outcomes.

## 1. Introduction

The anticoagulants used to prevent thromboembolism can be broadly divided into two categories: vitamin K-dependent antagonists (VKAs) and non-vitamin K oral anticoagulants (NOACs). The VKA warfarin has been employed as an anticoagulant for decades. However, NOACs are gaining popularity in clinical practice, replacing warfarin because of their excellent effectiveness [1,2,3].

Continued anticoagulation therapy, even when bleeding is controlled, can substantially elevate the risk of postoperative bleeding in oral surgery [4]. In 2020, the American College of Cardiology established a consensus on the management of bleeding in patients receiving anticoagulants, and in the dental community, clinicians proposed guidelines for managing perioperative bleeding during minor oral surgeries to ensure optimal care for patients [5,6,7].

To minimize the risk of bleeding and complications, the international normalized ratio (INR) is the preferred parameter for evaluating patients using VKAs. The INR varies considerably owing to factors such as the diet, gastrointestinal system, and amount of vitamin K-dependent clotting factors of the patient, as well as interactions with other medications. Although the INR must be closely monitored and adjusted to ensure it falls within the therapeutic range (2.0–3.0), checking the INR 72 h before oral surgery has practical limitations, including cost and time constraints. Many clinicians induce hemostasis without changing the regimen for perioperative bleeding control when the INR is below 3.5; however, more reliable indicators are required for preoperative evaluations [8,9].

Nonetheless, the scope of NOAC use is expanding. Recently, fixed-dose therapy was found to significantly reduce the risk of thromboembolism [10,11,12]. Furthermore, NOACs have relatively fewer interactions with dietary compounds or drugs when compared to VKAs. Moreover, in contrast to VKAs, NOACs do not require routine monitoring, owing to their reliable pharmacokinetic properties [13]. When managing perioperative bleeding, some clinicians skip medications or adjust the dosage, whereas others recommend maintaining the regimen. Therefore, there is no established consensus on managing postoperative bleeding in patients receiving anticoagulants.

Few studies have analyzed and compared the risk factors for postoperative bleeding in oral surgeries, including dental implant surgeries, between warfarin and NOACs. We hypothesized that in patients taking anticoagulants, distinct factors specific to each drug influence postoperative bleeding after oral surgery when anticoagulation therapy is not discontinued. Therefore, this study aimed to investigate the effectiveness of the predictors of bleeding complications in patients taking anticoagulants without altering their regimens when they underwent minor oral surgery.

## 2. Materials and Methods

### 2.1. Ethical Considerations

The need for informed patient consent was waived owing to the retrospective nature of the study. The study design was approved by the Ethics Committee of Ulsan University Hospital (IRB File No. UUH 2023-08-064).

### 2.2. Patients and Data Collection

We retrospectively reviewed the electronic medical and dental records of patients receiving either warfarin or NOACs who underwent oral surgery at the Ulsan University Hospital between January 2015 and August 2023. The only inclusion criterion was the use of warfarin or NOACs (Eliquis, Pradaxa, Xarelto, and Lixiana), and the patients were instructed not to stop taking or change their medication regimen before intraoral surgery.

We collected data from records that met the above criteria, including data on age, sex, warfarin/NOAC medication, drug indication, type of oral surgery, INR, time in the therapeutic range (TTR), and perioperative and postoperative bleeding outcomes. Patients with diseases affecting hemostasis and coagulation, such as von Willebrand disease and hemophilia, as well as those with an INR greater than 3.5 on the day of the procedure or with incomplete data, were excluded from the study.

Minor oral surgical procedures included dental procedures with the potential for bleeding, such as tooth extraction, flap operations, and implant surgery. All procedures were performed under local anesthesia by experienced oral and maxillofacial surgeons. The standard approach involved the use of mucoperiosteal flaps, and in some cases, minimal incisions were made. Tooth extractions, periodontal treatments, and implant placements were then carried out, with sutures applied in all cases.

Bleeding was categorized as either perioperative or postoperative. Perioperative bleeding was considered if hemostatic agents were used. Conversely, postoperative bleeding was defined as a return visit to the outpatient or emergency department for postoperative bleeding with the timing of the postoperative bleeding recorded.

Local hemostatic measures were applied to all patients immediately after surgery. The primary measure was to apply pressure with a bite swab and to close the wound with resorbable Vicryl^®^ 3-0, 4-0, and 5-0 (Johnson & Johnson Medical GmbH, Norderstedt, Germany). Additional hemostatic measures were used in cases of perioperative bleeding. For tooth extraction, an absorbable atelocollagen sponge (Ateloplug^®^, TRM Korea, Seoul, Republic of Korea) was placed in the extraction socket prior to suturing, and tranexamic acid (Cyklokapron^®^, Pfizer Pharma GmbH, Berlin, Germany) was administered to inhibit plasmin and aid in hemostasis. In cases of postoperative bleeding, one or more of the following measures were used: pressure with a bite swab, resuturing, reapplication of hemostatic agents or, if necessary, bipolar electrocoagulation to control bleeding.

For patients receiving warfarin therapy, the effectiveness of anticoagulant therapy was assessed based on the INR. Warfarin minimizes the risk of thromboembolism when the INR falls within the therapeutic range [14]. Accordingly, the INR was consistently regulated in patients on VKAs to ensure that it remained within this range. The TTR represents the duration that the INR of patients stays within the desired range. TTR was calculated using Rosendaal’s method [15], using the following formula: (no. of days in range)/(total number of monitored days). Essentially, the TTR was calculated as the ratio of the number of times the INR fell within the 2.0–3.0 range [16], based on the four most recent INR values obtained during anticoagulant therapy, with the day of surgery as the reference. The calculation included the INR on the day of the procedure and the prior INR measurements.

### 2.3. Statistical Analysis

Categorical variables were expressed as frequencies with percentages, and continuous variables were expressed as the means ± standard deviation. Comparisons between groups were performed using the chi-square test or Fisher’s exact test for categorical variables, and Student’s t-test was used for continuous variables, as appropriate. Data manipulation and statistical analyses were performed using SPSS software (version 26.0; IBM Corp., Armonk, NY, USA). Statistical significance was set at *p* < 0.05 for all tests.

## 3. Results

A total of 226 patients were initially assessed for eligibility, of which 206 patients were ultimately included in the study: 86 patients on warfarin and 120 patients on NOACs (36 on Eliquis, 24 on Lixiana, 17 on Pradaxa, and 43 on Xarelto). The remaining 20 patients were excluded based on the predefined inclusion and exclusion criteria. The baseline patient characteristics are summarized in Table 1. No significant differences were observed between the warfarin and NOAC groups regarding age, sex, type of surgery, or presence of postoperative bleeding. However, because warfarin is recommended as the standard treatment for patients with artificial heart valves, a statistically significant difference was observed in the primary indications for patients between the groups (*p* < 0.001).

Postoperative bleeding in the warfarin and NOAC group is summarized in Table 2. In the warfarin group, there were no significant differences in sex or type of surgery between patients with and without postoperative bleeding; however, there was a significant difference in age. The INR of patients without bleeding complications was 2.24 ± 0.65, while that of patients with bleeding complications was 2.62 ± 0.87, representing a statistically significant difference (*p* = 0.022). Moreover, the calculated TTR in patients with postoperative bleeding was 48.56 ± 27.11 and 65.17 ± 20.80 in patients without postoperative bleeding. The presence of bleeding based on the TTR was statistically significant (*p* = 0.002). In contrast to the warfarin group, no significant differences were observed in age, sex, or surgery type between patients with and without postoperative bleeding in the NOAC group. However, there were significant differences in bleeding complications depending on the type of NOAC taken (*p* = 0.007). The association between postoperative and perioperative bleeding was assessed in all groups, revealing a statistically significant association between the two variables (Table 2; *p* < 0.001).

The timing of return visits to the outpatient or emergency department for patients who experienced postoperative bleeding is summarized in Table 3. Patients were categorized according to whether they returned on the day of surgery, within three days, or more than three days postoperatively. The majority of patients in both the warfarin and NOAC groups received local hemostatic measures on the day of surgery, with fewer requiring follow-up within three days or more than three days after the surgery.

## 4. Discussion

According to a recent systematic review [4], bleeding complications following minor oral surgery in patients receiving anticoagulants are generally not severe. Additionally, studies have shown that although terminating or altering anticoagulant treatment has a minimal effect on bleeding complications, it can also result in fatal outcomes [17,18,19]. Therefore, as part of oral guidelines for patients taking anticoagulants, it is desirable to accurately determine the characteristics of the drugs being administered preoperatively and manage bleeding complications using effective predictive factors.

In line with research on this topic, Lee et al. [20] examined postoperative bleeding risks in anticoagulated patients undergoing dentoalveolar surgery. While their study identified key risk factors, it did not extensively analyze time in the therapeutic range (TTR) or perioperative bleeding events. In contrast, our study focuses specifically on minor oral surgeries and incorporates TTR and different types of NOACs as additional predictors. These differences provide deeper insights into postoperative bleeding management and refine anticoagulation strategies for oral surgery patients.

For patients on warfarin, the age was higher in the postoperative bleeding group compared to that in the group without postoperative bleeding (*p* = 0.02). Previous studies [21] have suggested that older age may be associated with hemostatic issues in patients taking warfarin, and this study confirms that older age may be associated with a higher risk of bleeding.

In oral surgery, an INR of ≤3.5 is recommended as the threshold at which effective hemostasis can be achieved with only local treatment without the occurrence of postoperative bleeding. However, one study reported that an INR greater than 3.8 did not significantly affect the frequency of bleeding [10]. Additionally, even if the INR is within the desired range, it may not remain stable for long [22]; therefore, fluctuating INR values may reduce the reliability of predicting bleeding complications. Consequently, it is vital to ensure that the INR is effectively maintained within the therapeutic range where the TTR lies.

The TTR is typically calculated from the INR using three different methods (Rosendaal, traditional, and cross-sectional). Obtaining consistent results regarding bleeding risk based on the TTR is challenging because of the differences in the assumptions and tolerances of each calculation method. Although it is difficult to determine the superiority of these methods, the Rosendaal method is the predominantly used method in the literature, which assumes a dynamic INR, unlike other methods that assume a static INR. Therefore, in this study, the Rosendaal method was used to calculate the TTR within the 2.0–3.0 INR target range.

TTR has been demonstrated to be closely associated with complications such as stroke, bleeding, and death. Moreover, if the TTR falls below 60%, it is considered poor INR control, potentially leading to high mortality and serious bleeding [23]. Although this study was limited to minor surgeries, the significant correlation between TTR and bleeding complications, as demonstrated in previous studies, suggests that TTR is a meaningful predictor of bleeding complications [24,25]. According to Rocha et al. [26], TTR was not significantly associated with bleeding, but the reason for the significant results in this study was that the INR was measured frequently to ensure that it accurately reflected the patient’s true anticoagulation status.

The pharmacokinetics of each NOAC drug must be considered. The reliable pharmacokinetic properties of NOACs do not require periodic monitoring, which serves as an important factor in managing bleeding complications. The peak plasma concentration time of warfarin is 48–72 h, whereas NOACs reach peak plasma concentrations more rapidly at 4 h or less. The half-life of warfarin is 36–42 h, whereas that of NOACs is shorter at approximately 12–17 h for dabigatran, 7–11 h for rivaroxaban, 8–15 h for apixaban, and 9–10 h for edoxaban [11,27,28]. Considering the time to reach the maximum blood concentration and short half-life, postoperative bleeding can be controlled by discontinuing the drug; however, this increases the risk of thromboembolism [11,17]. In this retrospective study, several patients were readmitted to the emergency department because of delayed postoperative bleeding. Among these, only one case required drug discontinuation to achieve hemostasis, whereas in all the other cases, hemostasis was achieved using local methods.

We found statistically significant differences in bleeding complications depending on the type of NOAC taken. Consistent with previous studies [29], the overall risk of bleeding during surgery was higher with Xarelto and lower with Eliquis in this study. These differences may be attributed to variations in pharmacokinetics, such as peak plasma concentration time, bioavailability, and elimination half-life. Therefore, when selecting an NOAC for oral surgery patients, clinicians should account for these pharmacokinetic differences and optimize perioperative bleeding management through close monitoring and appropriate hemostatic measures. 

Previous studies found no significant difference in bleeding complications between the patients administered warfarin and NOACs [30]. In dental surgery, only minimal bleeding occurs, and the surgical site is relatively small; thus, clot formation and hemostasis can be achieved using standard sutures without the addition of hemostatic agents. Regarding the dental surgeries performed in this study, there was no significant difference in the degree of bleeding between the two experimental groups. Previous studies have shown that perioperative bleeding is associated with postoperative bleeding in patients taking warfarin [26], and similarly, in this study, we found that perioperative bleeding was significantly associated with postoperative bleeding in both the warfarin and NOAC groups.

Postoperative bleeding can be classified into early and delayed postoperative bleeding based on its timing [31]. In the present study, no severe cases of postoperative bleeding were observed, and all cases were managed with additional hemostatic measures. The results of this study suggest that the risk of postoperative bleeding is highest immediately after surgery. This is likely due to the diminishing effects of hemostatic measures applied during or immediately after surgery, which may lead to re-bleeding. Therefore, it is important to inform patients of the potential risk of bleeding right after surgery to ensure timely management.

Patients receiving anticoagulation therapy often require multiple medications, increasing the risk of drug–drug interactions that may alter anticoagulant efficacy and safety. NOACs, particularly rivaroxaban and apixaban, are substrates of CYP3A4 and P-glycoprotein (P-gp) [27]. Strong CYP3A4 inhibitors (e.g., ketoconazole, clarithromycin) can elevate NOAC plasma concentrations, thereby increasing bleeding risk, whereas CYP3A4 inducers (e.g., rifampin, carbamazepine) can accelerate clearance, reducing anticoagulant effectiveness and increasing thromboembolic risk [32,33]. Warfarin is primarily metabolized via CYP2C9 [27], and its activity can be significantly altered by interactions with antibiotics and NSAIDs. Antibiotics can increase bleeding risk by disrupting intestinal bacteria responsible for vitamin K production, leading to reduced synthesis of vitamin K-dependent coagulation factors [34]. NSAIDs, including ibuprofen and naproxen, impair platelet function and disrupt gastrointestinal mucosa, compounding bleeding risk [35].

As a retrospective study, this study has several limitations. First, the relatively small sample size and number of bleeding outcomes could have introduced bias. Second, the factors regarding tooth and periodontal conditions that could affect bleeding complications were not assessed, and the type of oral surgery was not stratified according to the extent of surgery, which may have contributed to variability. Finally, this study identified key factors influencing postoperative bleeding; however, it did not account for all potential confounders, including variations in anticoagulant dosage and specific drug interactions in patients receiving multiple medications. Future research should incorporate detailed medication histories and stratify patients based on concurrent drug use to better assess their impact on anticoagulation outcomes.

## 5. Conclusions

Collectively, the results of this study underscored the relationship between postoperative bleeding and specific factors associated with each medication in patients taking warfarin or NOACs who did not discontinue their medication and underwent oral surgery. Identifying these predictors can enhance patient management strategies by enabling more effective preoperative and perioperative assessments of patients on anticoagulants, thereby reducing postoperative bleeding and optimizing surgical outcomes.

## Figures and Tables

**Table 1:** Patient demographics and characteristics between the two groups.

	Warfarin (*n* = 86)	NOAC (*n* = 120)	*p*-Value
Mean age ± SD (years)	68.83 ± 9.34	71.40 ± 7.47	0.058 ^a^
Sex, *n* (%)			
Male	49 (57.0)	77 (64.2)	0.296 ^b^
Female	37 (43.0)	43 (35.8)	
Primary indication for oral anticoagulant therapy, *n* (%)			
Artificial heart valve	24 (27.9)	0	<0.001 ^b,^*
Atrial fibrillation	50 (58.1)	104 (86.7)	
Deep vein thrombosis	3 (3.5)	9 (7.5)	
Pulmonary embolism	5 (5.8)	5 (4.2)	
Others	4 (4.7)	2 (1.6)	
Type of oral surgery, *n* (%)			
Extraction	43 (50.0)	69 (57.5)	0.07 ^b^
Flap operation	33 (38.4)	29 (24.2)	
Implant surgery	10 (11.6)	22 (18.3)	
INR	2.40 ± 0.77		
TTR	58.49 ± 24.79		
Postoperative bleeding, *n* (%)			
With postoperative bleeding	36 (41.9)	48 (40.0)	0.789 ^b^
Without postoperative bleeding	50 (58.1)	72 (60.0)	

* Significant difference between the two groups; *p*-value determined using ^a^ Student’s *t*-test or ^b^ chi-square test. SD—standard deviation; INR—international normalized ratio; TTR—time in the therapeutic range.

**Table 2:** Characteristics of warfarin and NOAC groups.

	Warfarin			NOAC		
	With Postoperative Bleeding	Without Postoperative Bleeding	*p*-Value	With Postoperative Bleeding	Without Postoperative Bleeding	*p*-Value
Mean age ± SD	70.69 ± 10.20	65.60 ± 10.57	0.02 ^a,^*	72.21 ± 7.40	73.08 ± 8.17	0.544 ^a^
Sex			0.268 ^b^			0.277 ^b^
Male, *n* (%)	18 (50.0)	31 (62.0)		28 (58.3)	49 (68.1)	
Female, *n* (%)	18 (50.0)	19 (38.0)		20 (41.7)	23 (31.9)	
INR ± SD	2.62 ± 0.87	2.24 ± 0.65	0.022 ^a,^*			
TTR ± SD	48.56 ± 27.11	65.17 ± 20.80	0.002 ^a,^*			
NOAC Type						0.007 ^b,^*
Eliquis, *n* (%)				13 (27.1)	23 (31.9)	
Lixiana, *n* (%)				8 (16.7)	16 (22.2)	
Pradaxa, *n* (%)				2 (4.2)	15 (20.8)	
Xarelto, *n* (%)				25 (52.1)	18 (25.0)	
Perioperative bleeding			<0.001 ^c,^*			<0.001 ^b,^*
Absent, *n* (%)	4 (8.9)	41 (91.1)		20 (24.4)	62 (75.6)	
Present, *n* (%)	32 (78.0)	9 (22.0)		28 (73.7)	10 (26.3)	
Type of oral surgery			0.201 ^c^			0.186 ^b^
Extraction, *n* (%)	22 (51.2)	21 (48.8)		30 (43.5)	39 (56.5)	
Flap operation, *n* (%)	10 (30.3)	23 (69.7)		13 (44.8)	16 (55.2)	
Implant surgery, *n* (%)	4 (40.0)	6 (60.0)		5 (22.7)	17 (77.3)	

* Significant differences between the two groups; *p*-values from ^a^ Student’s *t*-test, ^b^ chi-square test, and ^c^ Fisher’s exact test. SD—standard deviation; INR—international normalized ratio; TTR—time in the therapeutic range; NOAC—non-vitamin K oral anticoagulant.

**Table 3:** Timing of the postoperative bleeding in warfarin and NOAC groups.

	Warfarin	NOAC
On the day of surgery, *n* (%)	22 (61.1)	29 (60.4)
Up to three days, *n* (%)	8 (22.2)	13 (27.1)
More than three days, *n* (%)	6 (16.7)	6 (12.5)
Total, *n* (%)	36 (100.0)	48 (100.0)

NOAC—non-vitamin K oral anticoagulant.

## Data Availability

The data are included in the study, further inquiries can be directed to the corresponding author.

## Abbreviations

The following abbreviations are used in this manuscript:

INR	international normalized ratio
NOACs	non-vitamin K oral anticoagulants
SD	standard deviation
TTR	time in the therapeutic range
VKA	vitamin K-dependent antagonists
